# Knowledge, Attitude and Practices (KAP) of Medical Professionals on Euthanasia: A Study From a Tertiary Care Centre in India

**DOI:** 10.7759/cureus.34788

**Published:** 2023-02-08

**Authors:** Raghvendra S Shekhawat, Tanuj Kanchan, Ashish Saraf, Navneet Ateriya, Vikas P Meshram, Puneet Setia, Mohini Rathore

**Affiliations:** 1 Forensic Medicine, All India Institute of Medical Sciences, Jodhpur, Jodhpur, IND; 2 Forensic Medicine and Toxicology, All India Institute of Medical Sciences, Jodhpur, Jodhpur, IND; 3 Forensic Medicine and Toxicology, All India Institute of Medical Sciences, Gorakhpur, Uttar Pradesh, India, Gorakhpur, IND; 4 Forensic Medicine, All India Institute of Medical Sciences, Gorakhpur, Gorakhpur, IND; 5 Biochemistry, All India Institute of Medical Sciences, Jodhpur, Jodhpur, IND

**Keywords:** euthanasia, mercy killing, physician assisted suicide, active and passive euthanasia, medical profession

## Abstract

Introduction: Euthanasia or mercy killing has remained as a matter of extensive debate and ethical conflicts in the scientific literature. Discussions on this theme have got legal, religious, political and philosophical ramifications.

Aim of the study: The present study aimed to assess the knowledge, attitude and practices of medical professionals in a tertiary care hospital.

Methods: After taking prior approval from the institutional ethics committee a structured questionnaire was prepared and distributed among 200 consenting medical professionals in a tertiary care centre in the North-Western region of India.

Results: 50% of the respondents said that they were sure of the existing regulation on euthanasia in India. When gender differences were considered a significant difference (p=0.0147) was found between the two sexes regarding the alternate decision maker for deciding euthanasia. It was observed that there was a significant difference(p=0.0055) between those with the age more than 30 years and aged less than 30 years regarding the type of euthanasia that is justifiable.

Discussion: In the present study, the percentage of doctors favouring euthanasia is higher than compared in previous studies. The view of euthanasia is highly variable in different studies. Even though passive euthanasia has been legalised recently, there is an apprehension that it might be misused.

## Introduction

Euthanasia has remained a matter of extensive debate and ethical conflicts in the scientific literature. Discussions on this theme have got legal, religious, political and philosophical ramifications [1]. Conventionally euthanasia has been classified into its active and passive forms, although such definitions suffer from criticism by various authors. In the active type, the person performing the euthanasia does an act that leads to the death of the individual like injecting a lethal drug, whereas, in the passive type, life support is either withdrawn or withheld, e.g., withdrawal of ventilator support. For the sake of understanding and categorization, it can be held that in active euthanasia there is an act of commission and in the passive type, there is an act of omission [2,3]. The concept of euthanasia essentially revolves around themes like ‘human dignity', ‘patient autonomy', and ‘the beneficence of the subject'. The decision maker chooses for euthanasia as a last resort because he/she believes that ending the life is a better decision than prolonging a painful and unfruitful life. The countries having discreet and specific mention of euthanasia in their legislature mostly concentrate on the passive form. There are a few countries where active euthanasia has got legal sanctioning, e.g., the Netherlands [4].

In India, the issue of euthanasia gained momentum after the Aruna Ramchandra Shanbaug v. Union of India and others case wherein a human rights activist petitioned the Supreme Court in 2009 and pleaded that artificial feeding is stopped to let her die in dignity [5]. The court rejected the plea of the activist but admitted that there is an enormous need to frame guidelines for a vast country like India. Interestingly, while referring to the Airedale NHS Trust v. Bland case the court discussed the doctrine of Parens Patriae with respect to Indian laws and highlighted the need for surrogate decision-makers in case the patients were incompetent to make a decision. In a phase when lawmakers are proactively introducing new concepts to the theme of euthanasia in a densely populated country like India, it becomes pertinent and indispensable for medical professionals to make themselves aware of these changes. The present study aimed to assess the knowledge, attitude and practices of medical professionals in a tertiary care hospital.

## Materials and methods

Prior approval was taken from the institutional ethics committee for conducting the study. The questionnaire was prepared was validated with the help of an expert committee followed by a pilot test on 10 subjects. The questionnaire was then prepared was distributed physically to the doctors working in the tertiary health care centre where the study was performed. The questionnaire was distributed among 200. (This number was selected based on the availability of a number of doctors in the tertiary care centre during the study period.) consenting medical professionals (residents and faculty) in a tertiary care centre in the North-Western region of India. Informed consent was taken at the time of handing over the questionnaire. Those who refused to give consent were not handed over the questionnaire. Preliminaries like age, gender, religion, marital status, experience, qualifications, etc. were sought. The questionnaire was aimed at assessing the perceptions, knowledge, and attitude of the physicians towards euthanasia, both active and passive forms. Information regarding the practice of euthanasia was also collected. The data thus obtained from the questionnaire was analysed using SPSS 26.0. The responses were also analysed to observe responses among males and females and those aged below and above 30 years of age. This age was selected with the assumption that; this is the age when the responsibilities of the family are managed significantly in the Indian context. Persons aged less than 30 years are usually free from family responsibilities. A chi-square test was applied to look for age and gender differences in the responses. A p-value less than 0.05 was considered significant. The total duration of the study was about four months, and it was conducted in the year 2019. Inclusion criteria- All doctors who were working in the tertiary care centre during the study period. Exclusion criteria- All who did not want to participate and refuse to give consent for the study.

## Results

The respondents were aged between 23 years and 58 years with a mean age of 32.34 ± 5.88 years. The experience of the respondents ranged between three months and 37 years with the mean experience being 4.95 ± 5.44 years. The majority of the respondents were married males belonging to the Hindu community. Around 71% of the respondents were medical postgraduates, and about 65% of them were resident doctors who were the first point of contact for the patient. Details of the descriptive statistics are shown in Table 1. For questions related to awareness about euthanasia, 50% of the respondents said that they were sure of the existing regulation on euthanasia in India. Awareness regarding the legal status of the type of euthanasia was also enquired where 7% of them believed that the active type is legalised and about 47% believed that the passive type is legalised (Table 2). The attitude of participants towards euthanasia in general, and that based on their gender and age is depicted in Tables 3, 4. Opinions regarding the alternate decision maker and the reason for supporting or opposing euthanasia were also sought, the details of which are shown in Tables 5-7. Most of the respondents selected from the options available in the questionnaire for why they supported or opposed euthanasia. However, three respondents had other reasons to offer too; the additional reasons for supporting euthanasia were, “if the patient is critically ill in and life-threatening condition” and “for organ derivation in severely ill patient”, while the additional reason for opposing euthanasia was that the “regulation will become difficult if legalised”. Table 8 shows the responses of the participants regarding the practices relating to euthanasia.

**Table 1:** Descriptive statistics of the study sample (N=200)

Age (Years)	Range	23.0-58.0
	Mean ± SD	32.3 ± 5.9
Experience (Years)	Range	0.25 - 37.0
	Mean ± SD	4.9 ± 5.4
Gender N (%)	Male	147 (73.5)
	Female	53 (26.5)
Marital Status N (%)	Married	136 (68.0)
	Unmarried	64 (32.0)
Religion N (%)	Hindu	192 (96.0)
	Muslim	05 (2.5)
	Sikh	02 (1.0)
	Buddhist	01 (0.5)
Qualification N (%)	Medical Graduate	42 (21.0)
	Medical Post-Graduate	142 (71.0)
	PhD	08 (4.0)
	Dental Postgraduate	08 (4.0)
Designation N (%)	Professor	07 (3.5)
	Additional Professor	06 (3.0)
	Associate Professor	17 (8.5)
	Assistant Professor	39 (19.5)
	Senior Resident	90 (45.0)
	Junior Resident	41 (20.5)
Department N (%)	Pre-Clinical	25 (12.5)
	Para-Clinical	46 (23.0)
	Clinical	129 (64.5)

**Table 2:** Response to prevalent legal status about the type of euthanasia among the participants

Item	Legal, N (%)	Illegal, N (%)	Not sure, N (%)
Status of Active Euthanasia in India?	14 (7.0)	181 (90.5)	05 (2.5)
Status of Passive Euthanasia in India?	95 (47.5)	100 (50.0)	05 (2.5)
Status of Physician-Assisted Suicide in India?	14 (7.0)	175 (87.5)	11 (5.5)

**Table 3:** Response (sex and age based) to view regarding the type of euthanasia that is justifiable

	Total, N (%)	Male, N (%)	Female, N (%)	Age (≤ 30 years), N (%)	Age (> 30 years), N (%)
Active	17 (8.5)	12 (6.0)	05 (2.5)	13 (6.5)	04 (2.0)
Passive	81 (40.5)	61 (30.5)	20 (10.0)	37 (18.5)	44 (22.0)
Both	67 (33.5)	53 (26.5)	14 (7.0)	21 (10.5)	46 (23.0)
None	24 (12.0)	16 (8.0)	08 (4.0)	08 (4.0)	16 (8.0)

**Table 4:** Response (sex and age based) to views regarding the type of euthanasia that is acceptable

	Total, N (%)	Male, N (%)	Female, N (%)	Age (≤ 30 years), N (%)	Age (> 30 years), N (%)
Voluntary	109 (54.5)	78 (39.0)	31 (15.5)	49 (24.5)	60 (30.0)
Non-Voluntary	08 (4.0)	07 (3.5)	01 (0.5)	02 (1.0)	06 (3.0)
Both	44 (22.0)	34 (17.0)	10 (5.0)	18 (9.0)	26 (13.0)
None	32 (16.0)	23 (11.5)	09 (4.5)	12 (6.0)	20 (10.0)

**Table 5:** Response (sex and age based) to views regarding whom should be the alternate decision maker for deciding euthanasia

	Total, N (%)	Male, N (%)	Female, N (%)	Age (≤ 30 years), N (%)	Age (> 30 years), N (%)
Treating doctor	85 (42.5)	55 (27.5)	30 (15.0)	36 (18.0)	49 (24.5)
Next of kin	55 (27.5)	49 (24.5)	06 (3.0)	19 (9.5)	36 (19.0)
Court order	53 (26.5)	40 (20.0)	13 (6.5)	22 (11.0)	31 (15.5)
Patient's lawyer	08 (4.0)	06 (3.0)	02 (1.0)	03 (1.5)	05 (02.5)

**Table 6:** Response (sex and age based) to views regarding why the respondents support euthanasia

	Total, N (%)	Male, N (%)	Female, N (%)	Age (≤30 years), N (%)	Age (>30 years), N (%)
Humane to end life of prolonged suffering	151 (75.5)	115 (57.5)	36 (18.0)	61 (30.5)	90 (45.0)
Favour individual right to die with dignity	105 (52.5)	74 (37.0)	31 (15.5)	35 (17.5)	70 (35.0)
Prevent unnecessary waste of medical resources	39 (19.5)	29 (14.5)	10 (5.0)	17 (8.5)	22 (11.0)
Lessens unnecessary financial burden on relatives	68 (34.0)	57 (28.5)	11 (5.5)	30 (15.0)	38 (19.0)
Others	02 (1.0)	02 (1.0)	0 (0.0)	02 (1.0)	0 (0.0)

**Table 7:** Response (sex and age based) to views regarding why the respondents oppose euthanasia

	Total, N (%)	Male, N (%)	Female, N (%)	Age (≤30 years), N (%)	Age (>30 years), N (%)
Against medical ethics of saving life	54 (27.0)	44 (22.0)	10 (5.0)	26 (13.0)	28 (14.0)
Due to one’s religious/cultural beliefs	12 (6.0)	07 (3.5)	05 (2.5)	04 (2.0)	08 (4.0)
Availability of good palliative care	36 (18.0)	24 (12.0)	12 (6.0)	15 (7.5)	21 (10.5)
Can be misused for personal gains	125 (62.5)	92 (46.0)	33 (16.5)	46 (23.0)	79 (39.5)
Others	01 (0.5)	01 (0.5)	0 (0.0)	01 (0.5)	0 (0.0)

**Table 8:** The practice of euthanasia among the participants

Item	Yes, N (%)	No, N (%)	Not answered, N (%)
Do you encounter terminally ill patients in your practice?	119 (59.5)	80 (40.0)	01 (0.5)
Any terminally ill patient ever requested you to end his/her life?	48 (24.0)	150 (75.0)	02 (1.0)
Critically ill patient’s relative/s ever requested you to end his/her life?	37 (18.5)	160 (80.0)	03 (1.5)
Have you ever discussed euthanasia with critically ill patients/relatives?	27 (13.5)	171 (85.5)	1.0)

When gender differences were considered a significant difference (p=0.0147) was found between the two sexes regarding the alternate decision maker for deciding euthanasia (Table 5). No significant gender differences were observed for the other issues in question. It was observed that there was a significant difference(p=0.0055) between those the aged more than 30 years and aged less than 30 years regarding the type of euthanasia that is justifiable (Table 3). No significant differences were observed for the other issues in question between those aged less than 30 years and more than 30 years.

## Discussion

According to clause 6.7 of Professional Conduct, Etiquette and Ethics Regulations, 2002 of the Medical Council of India, practicing euthanasia accounts for unethical conduct [6]. The World Medical Association in its declaration on euthanasia has stated that “Euthanasia, that is the act of deliberately ending the life of a patient, even at the patient’s request or at the request of close relatives, is unethical”. This was adopted way back in the 39th World Medical Assembly in 1987 and was reaffirmed in 2005 and 2015 [7]. The Indian Society of Critical Care Medicine (ISCCM) in 2012 has framed “Guidelines for limiting life-prolonging interventions and providing palliative care towards the end of life in Indian Intensive care units” in section 3 of ICU Guidelines [8]. ISCCM is a non-profit association of Indian Physicians, Nurses, Physiotherapists, and other allied healthcare professionals involved in the care of the critically ill. The ISCCM has been making continuous efforts for framing and updating the guidelines on euthanasia. The 241st report of the Law Commission of India published in August 2012 recommended that passive euthanasia shall have legal recognition in India and is not objectionable from a legal and constitutional point of view. It was mentioned that a competent patient who is above the age of 16 years has the right to insist that there should be no use of artificial life-sustaining measures or treatment which shall be binding on the doctors/hospital provided that the doctor is satisfied that the patient has taken an informed decision based on free will. In the case of an incompetent patient, the doctor's or relative's decision to withhold or withdraw the medical treatment is not final, and the same can be done after getting clearance from the High Court. It also highlighted that the advance medical directive given by the patient before his illness is not valid [9].

Recently in March 2018, the Supreme Court of India has taken a call to frame a prudent legislature on passive euthanasia and has laid down guidelines. It said that an adult human has the right to refuse medical treatment including withdrawal from life-saving devices provided he has the mental capacity to take the decision. The court also allowed the execution an advanced health directive by a person of the competent mental faculty [10].

There are only a few countries that have legalised euthanasia. Most of the countries have legalised the passive form [4]. There is a minority of them who have legally approved active euthanasia and physician-assisted suicide [4]. Medical Professionals like doctors and nurses face a dilemma and have a tough time in decision-making with regard to euthanasia while dealing with patients and/or their relatives. Doctor's view regarding euthanasia is vital in deciding if euthanasia should be allowed or not as he/she is well aware of the condition of the patient, the prognosis of the disease, and the suffering which the patient might be suffering from.

In our study, 80% of the participants were of the opinion that euthanasia should be allowed for a terminally ill patient. This is in contrast to the previous studies where only 19% [11], 27% [12], 43% [13,14], 47% [15], and 69% [1] favoured euthanasia. However, similar results were observed by Marc Helt et al. [16] wherein 84% favoured euthanasia. These variations may be attributed to the different study populations, period of study, the methodology followed, and the legal scenario in the country where the study was done. In the present study, the most common reason why they were in favour of euthanasia was that they believed it was "humane to end a life of prolonged suffering" followed by the realise that every "individual has the right to die with dignity". Similar observations were made by Kamath et al. [1], Abbas et al. [12] and Sonu et al. [15]. In the current study participants, a significant difference (p=0.0055) of opinion was observed regarding the type of euthanasia that is justifiable between those aged more than 30 years and those less than 30 years. This difference might be due to the fact that those less than 30 years must not have encountered a chronic disease in their lifetime. However, those aged more than 30 years might have come across a chronic disease that might have changed their opinion.

It is also worth observing that although euthanasia had no legal status during the study period, there were about 25% of the doctors who had received a request for euthanasia by a terminally ill patient and about 18% of the doctors who had received a request for euthanasia by a relative of a terminally ill patient. This implies that there might be a good number of people/surrogate decision-makers who favour euthanasia for some or the other reason. In our study, most of the respondents believed that the treating doctor followed by the next of kin should be the alternate decision maker in a case of non-voluntary euthanasia. Similar observations were made by Kamath et al. [1]. In the present study, a significant difference of opinion was observed with regard to the alternate decision maker for deciding euthanasia.

This difference of opinion might be attributed to the fact that India is mostly a patriarchal society, and usually, the males manage the financial aspects of the family and hence the difference in opinion. There was no other significant difference in opinion when both genders were considered. Similar results were also obtained in studies by Kamath et al. [1], Singh et al. [17], Adesina et al. [18] and Anneser et al. [11]. A study by Vijaylakshmi et al. however, observed significant male-female differences in responses to various issues relating to euthanasia [19].

## Conclusions

In the present study, the percentage of doctors favouring euthanasia is higher than compared in previous studies. The view of euthanasia is highly variable when different studies are compared. The variability may be attributed to religious beliefs, their practical experience with critically ill patients, etc. Even though passive euthanasia has been legalised recently, there is an apprehension that it might be misused. The limitation of the present study is its small sample size from a tertiary hospital that may not be representative of the medical professionals in the country. Hence, similar studies on larger samples are proposed.
